# General Pathology and Treatment of Dyspepsia

**Published:** 1854-03

**Authors:** J. H. Bennett

**Affiliations:** The University of Edinburgh


General Pathology and Treatment of Dyspepsia.—By Prof. J. H. Bennett, M. D., of the University of Edinburgh. By Dyspepsia is generally understood all those functional derangements of the stomach which are primary in their origin, that is, not dependent upon, nor symptomatic of, inflammation or other disease in the economy. Such a disordered condition is exceedingly common, and often constitutes the despair of the physician, arising as it frequently does, from causes which are often obscure, or if discovered, are beyond his control. This will become apparent by considering, in the first place, those circumstances which require to be united to secure a healthy digestion. These are:—1st. A proper quantity and quality of the ingesta. 2d. Sufficient mastication and insalivation. 3d. Active contractility in the muscular coat of the stomach. 4th. Proper quantity aud quality of the gastric, biliary, and pancreatic fluids. 5th. A consecutive and harmonious action of the intestinal canal. Dyspepsia, or indigestion may be produced by any cause which occasions derangement of one or more of these conditions; and hence why so many different circumstances may produce somewhat similar symptoms, and why so many different remedies have been found effectual in various cases. Notwithstanding that you will frequently meet with instances which baffle all preconceived rules, there can be no doubt that a careful attention to the essential physiological conditions above enumerated, will in the great majority of cases, conduct you to a successful rational treatment. Thus,—

1st. Of all the causes of dyspepsia, excesses in eating and drinking are the most common. An over-distended stomach, or too rich a meal, not unfrequently induces a feeling of weight or fullness in the epigastrium, nausea and eructation of acid, bilious or gaseous matters, with a loaded tougue, headache, and other general symptoms. This is acute dyspepsia, or the *embarras gastrique* of the French. Occasionally, there is more or less vomiting of bilious matter, when the attack is vulgarly called a *bilious seizure.* If called in to such a case, immediately on its occurrence, and before the ingesta have left the stomach, as determined by the sense of load at the epigastrium, an emetic should be given; but if vomiting be present, it should be assisted by warm diluents. As soon as the stomach is quieted, or, if you have been called in at a late period, when the ingesta have passed into the intestines, a purgative pill should be administered, consisting of four grains of calomel, with four of compound extract of colocynth, followed in a few hours by a purgative draught of salts and senna. If necessary also an enema may be given. The purging, with a day or two's confinement to farinaceous food, will generally get rid of such an attack; but their frequent repetition leads to the chronic form of dyspepsia, when careful regulation of the diet with exercise, must constitute the chief treatment. Hence the advantage of what is called "change of air," and much of the benefit which is derived from watering-places. Chronic dyspepsia, however, is far more commonly caused by excess of spi.ituous and vinous drinks, than by eating, when abandonment of the evil habit is a *sine qua non* in the treatment. Tea drinkers are very liable to the disease, and its frequency among female servants is probably owing to this cause.

2d. It may frequently be noticed, that those who have acquired the habit of eating rapidly are more or less dyspeptic. I knew a journeyman printer who was much tormented with indigestion, but who was cured by changing his residence. The cause of this was for some time a mystery, but on again changing his house the disease returned, although no apparent cause could be discovered. I ascertained, however, that it depended not on the locality *per se*, but on its distance from the printing-house. When far off he ate his dinner with his family rapidly, having only just time enough to walk home and back within the hour. When he lived near, the time otherwise spent in walking was occupied in eating, or in cheerful converse with his wife and family. Since I made this observation, it has often occurred to me that the distant residences of artisans from their place of employment may be the occasional cause of the dyspeptic symptoms they frequently possess. With regard to the exact object of the saliva in the process of digestion, whether it be to convert the farinaceous compounds of the food into glucose, or by its viscidity, to mix up air with the portions swallowed, is not positively determined; but its necessity for the digestion of man is shown by cases where the under lip has been lost by accident or disease, and where salivary fistula has formed, in which dyspepsia is generally present, and in which the disordered digestion has been cured by operations that, by restoring the parts to their normal condition, prevent the escape of saliva. Again, persons habituated to the dirty habit of spitting, are for the most part dyspeptic; and it has been asserted that the pale countenances of the inhabitants of the United States and the leanness of their persons are owing to this cause. In all cases where dyspepsia can be traced to this source, the treatment must be obvious.

3d. The contractile movements of the stomach, which, by kneading the ingesta, and keeping them in constant motion, secures an intimate admixture with the gastric juice and the rapid transference to the duodenum of such portions of it as are transformed into chyme, are evidently of immense importance to a proper performance of digestion. The experiments of physiologists have shown that digestion in gastric juice out of the stomach is much slower than in it, and that section of the pneumogastris nerves, by arresting the contractile movements, only permits the circumference of the mass in contact with the secreting surface to be digested.—These facts at once explain the well-known influence of mental emotions upon the stomach. Contentment and hope are as favorable, as dissatisfaction and despondency are opposed to good digestion. Nothing is more common than dyspepsia among literary men who overtask the mental faculties; among young persons of very excitable minds; and among individuals of a melancholy temperament, hypochondriacs, etc., etc. It is in such cases that cheerful society, active and appropriate occupations, change of scene, removal from mercantile or literary employments, different trains of thought, and so on, are beneficial. Hence also many of the good effects of travel, visiting watering-places, &c., &c.

4th. Our knowledge with regard to the offices performed by the gastric, biliary and pancreatic juices in digestion has of late years been much advanced. Thus the gastric juice more especially operates on the albuminous, and the pancreatic juice on the fatty compounds of the food. The function of the bile is perhaps more obscure, although it probably acts as a means of precipitating or separating some of the excretory matters from chyme, and so facilitates assimilation of the nutritive portions. Digestion may be deranged by all those causes which too much increase or diminish the secretion of these three fluids. Thus excess of acidity in the stomach is one of the most common causes of dyspepsia, producing that form of it which accompanies scrofulous and tubercular diseases. It may be in such excess as to neutralize the alkaline action of the pancreatic juice, and render it difficult or impossible to emulsionize fatty matters. In such cases, alkalies, with bitter tonics, and the direct introduction of animal oils in excess, are indicated. On the other hand, the gastric juice may be diminished in quantity, as frequently occurs in persons who suddenly overtask the powers of the stomach at feasts, or in old persons with feeble digestion. The sense of load after eating is generally indicative of slow digestion from this cause. In acute cases, a stimulant rouses the stomach to increased action, and hence the moderate use of drams and generous wines after dinner is occasionally useful. In old persons the sense of load and feebleness is best removed by giving up tea, and drinking at night a little weak brandy and water. In chronic cases, acids are indicated, especially muriatic acid. The *Tr. Ferri co.* of the Pharmacopœia is a useful preparation in chlorotic females. We have no distinct means, as far as I am aware, of rousing the pancreas into into action, and yet many cases are on record in which fatty matters have passed undigested through the alimentary canal in consequence of obstruction to the pancreatic duct. In such cases, and all those in which fatty matters are difficult to digest, alkalies, especially the liquor potassæ with vegetable tonics, are indicated. When the bile is deficient, constipation and dyspepsia are usual results, and are to be relieved by gentle mercurial purgatives, with extract of taraxacum, and by remedies, such as rhubarb and especially the compound rhubarb pill, which, by acting on the duodenum, also favor the flow of bile into the upper part of the alimentary canal. Dr. Clay, of Manchester, has recommended in such cases the administration of ox-gall, a remedy, which, although not extensively given, is evidently rational, and calculated by its purgative action to be highly serviceable. Excess of bile, on the other hand, ought to be treated by drastic purgatives, diuretics and diaphoretics, according to circumstances, to cause excess of excretion. Exercise should also be insisted on to call the lungs into action, and thus relieve the liver in its office of separating hydro-carbon.

5. A derangement of the consecutive and harmonious action of the alimentary canal is another frequent cause of dyspepsia, for it is as necessary that those portions of the food which are not assimilable should be removed out of the economy, as that the nutritive materials should be absorbed. Hence whatever impedes the contractility of the intestinal canal, whatever alters the structure of its mucous membrane, or whatever mechanically obstructs its calibre, may always be observed to induce dyspeptic symptoms. The removal of these various conditions, whether by stimulating the nervous centres, by appropriate diet, or by purgatives and astringents, as they may be required, need not be more particularly dwelt upon.

In many cases of dyspepsia, two or more of these causes may be combined, so as to render the indications for treatment complex and apparently contradictory. In other cases, one or more causes may exist, although from the indications presented they cannot be determined, when our treatment must always be more or less vague and unsatisfactory. Lastly there are a few instances where dyspepsia can only be explained by *idiosyncrasy*, in which we find this or that particular article of diet to derange the digestive functions, and in which avoidance of the offending cause is the only plan that is attended with success.—*Monthly Journal.*

